# The importance of waist circumference and body mass index in cross-sectional relationships with risk of cardiovascular disease in Vietnam

**DOI:** 10.1371/journal.pone.0198202

**Published:** 2018-05-29

**Authors:** Nga Thi Thu Tran, Christopher Leigh Blizzard, Khue Ngoc Luong, Ngoc Le Van Truong, Bao Quoc Tran, Petr Otahal, Mark Nelson, Costan Magnussen, Seana Gall, Tan Van Bui, Velandai Srikanth, Thuy Bich Au, Son Thai Ha, Hai Ngoc Phung, Mai Hoang Tran, Michele Callisaya

**Affiliations:** 1 Menzies Institute for Medical Research, University of Tasmania, Hobart, Tasmania, Australia; 2 Can Tho University of Medicine and Pharmacy, Can Tho City, Vietnam; 3 Medical Services Administration, Ministry of Health of the Socialist Republic of Vietnam, Ha Noi, Vietnam; 4 Department of Medicine, Peninsula Clinical School, Central Clinical School, Monash University, Melbourne, Victoria, Australia; Nagoya University, JAPAN

## Abstract

**Background:**

Waist circumference (WC) is an indicator of intra-abdominal adipose tissue, high levels of which confer an increased risk of cardiometabolic disease. Population data on WC should be more informative than data on body mass index (BMI), which is a general indicator of body size. This study aimed to evaluate the importance of WC relative to BMI in cross-sectional relationships with blood pressure (BP), glucose, and total cholesterol (TC) in the adult population of Vietnam.

**Methods:**

The data were collected in a population-based survey conducted during 2009–10 using the “WHO STEPwise approach to surveillance of risk factors for non-communicable disease” (STEPS) methodology. The survey participants (n = 14 706 aged 25 to 64 years) were selected by multi-stage stratified cluster sampling from eight provinces representative of the eight geographical regions of Vietnam. All measurements were performed in accordance with the STEPS protocols. All analyses were performed using complex survey methods.

**Results:**

The measurements of WC and BMI were highly correlated (men r = 0.80, women r = 0.77). For men, the strongest and predominant associations with BP, glucose, and TC were for WC or an index based on WC. For women, this was true for glucose but BMI was more important for BP and TC. WC or an index based on WC provided better discrimination than BMI of hypertension and elevated glucose, and of raised TC for men. Information on four new anthropometric indices did not improve model fit or subject discrimination.

**Conclusion:**

For BP/hypertension, glucose/elevated glucose, and TC/raised TC, WC was more informative than BMI for Vietnamese men, but both WC and BMI were important for Vietnamese women. Both WC and BMI need to be assessed for estimation of CVD risk in Vietnam.

## Background

Overweight and obesity is a predictor of the morbidity and mortality from cardiovascular diseases (CVD), diabetes, musculoskeletal disorders and some cancers [1, 2]. In addition, excess abdominal obesity is associated with a range of metabolic abnormalities and CVD [3, 4]. Body mass index (BMI) is widely used in the diagnosis of overweight and obesity, whereas waist circumference (WC) and indices based on WC–such as waist-to-hip ratio (WHR), and waist-to-height ratio (WHtR)–are employed as surrogate indicators of visceral obesity to predict morbidity and mortality at the population level [5–7]. These anthropometric indices are used in epidemiological studies for population surveillance of risk factors for chronic disease [8] because they can be easily measured and at a low cost [9].

In Western populations, there is not universal agreement on which measure, BMI or WC, is the more important predictor for chronic disease, particularly CVD. The consensus opinion is that BMI and WC provide different information for prediction of disease risk [7, 10]. In Asian populations, CVD prevalence has been found to increase continuously with BMI in studies that measured BMI only [11, 12]. However, there is emerging evidence that WC may be more important than BMI in predicting chronic disease including diabetes in Asian populations [13, 14].

Using data from a nationally-representative population-based survey of risk factors for CVD in Vietnam, this study aimed to examine the relative and combined contribution of WC and BMI for the estimation of blood pressure (BP) and hypertension, glucose and elevated glucose, total cholesterol (TC) and raised TC in the Vietnamese population and to identify which factor (BMI or WC) provides better discrimination of CVD risk. We also had the opportunity to investigate whether newly-proposed indices such as Body Adiposity Index (BAI) [15], Abdominal Volume Index (AVI) [16], Conicity Index (Cindex) [17], and A Body Shape Index (ABSI) [18] offer any improvement over BMI and WC.

## Methods

### Study participants and sampling

The survey participants, 25 to 64-year-old persons from eight provinces representative of the eight geographical regions of Vietnam, were selected by multi-stage stratified cluster sampling. In brief, two-stage cluster sampling was used to select 20 clusters (communes, towns, and city wards) from each of eight geographically-representative provinces with probabilities proportional to population size from four strata defined by urban–rural location and rich–poor classification. For each selected cluster, the provincial health authority prepared a comprehensive list of 25–64 years old residents. From those lists, 25 persons per cluster were selected in each age group (25–34 years, 35–44 years, 45–54 years, 55–64 years) and with approximately equal members of men and women. A total of 14 706 respondents (64.1% of all eligible people) participated in the survey. Data were collected during 2009–10 using the “WHO STEPwise approach to surveillance of risk factors for non-communicable disease” (STEPS) methodology [19]. Clinics were conducted in the local health station of each participant’s area of residence. Interviewers were staff of the provincial health authorities who were trained in the implementation of the STEPS methodology. Training of field staff was conducted pre-survey at training centres in Ha Noi, Hue and Ho Chi Minh city, and on-site at regular intervals by local, national and international supervisors. Eligible persons were invited to attend the clinic on a specific date, each clinic commencing in the early morning because overnight fasting was required. Data were collected and entered by trained staff of each provincial health authority. They underwent intensive training and supervision provided by the Menzies Institute for Medical Research, Australia. A pilot study was conducted to test survey instruments and procedures before actual data collection. All measurements were performed in accordance with the STEPS protocols. The study was approved by the Ethics Committee of Vietnam Ministry of Health and the Tasmanian Health and Medical Human Research Ethics Committee. Written informed consent was obtained from participants before collecting data.

### Measurements

Socio-demographic information and measurements of four behavioural factors (tobacco smoking, alcohol, fruit/vegetable consumption, and physical activity) were obtained using the STEPS questionnaire [19]. The questionnaire was translated into Vietnamese and back-translated to check the accuracy of wording of each item. Physical measurements included weight (in bare feet without heavy clothing measured using NuWeigh B8271 digital scales with the precision of 0.1 kg), height (in bare feet without headwear measured using a Seca 214 stadiometer with the precision of 0.1 cm), WC (at the level of the mid-point between the inferior margin of the last rib and the iliac crest measured horizontally using a constant tension tape while standing), and hip circumference (at the greatest posterior protuberance of the buttocks measured using a constant tension tape while standing). With weight expressed in kilograms (kg), height expressed in metres (m) and girths expressed in centimetres (cm), we calculated BMI as weight÷height^2^, WHR as WC÷Hip, WHtR as WC÷(height×100), BAI as (Hip÷100)÷(height^1.5^)–18, AVI as [2×WC^2^+0.7×(WC–Hip)^2^]÷1000, CIndex as $(\text{WC}÷100)÷0.109\times \sqrt{\text{weight}÷\text{height}}$, and ABSI as $(\text{WC}÷100)÷\text{BM}{\text{I}}^{2÷3}\times \sqrt{\text{height}}$.

BP was measured using an Omron HEM 907 digital automated BP monitor after participants had rested for at least 15 minutes. Two blood pressure readings taken 3 minutes apart were obtained for all participants. The protocol stipulated a third reading to be taken if there was a difference between the two readings of more than 25 mmHg for systolic blood pressure or more than 15 mmHg for diastolic blood pressure. For BP measurement, if a third measure was taken, the mean of the two closest measures was used; otherwise, the mean of the two measures was used. After overnight fasting, blood glucose and TC were measured from capillary whole blood using Roche Diagnostics Accutrend Plus glucometers. For BP and fasting glucose, participants were excluded if they reported taking medication to lower BP or for diabetes respectively. Raised BP or hypertension was defined as systolic BP ≥140 mmHg and/or diastolic ≥ 90 mmHg or currently on medication for raised BP. Elevated blood glucose was defined as capillary whole blood glucose ≥ 6.1 mmol/L or taking medications for raised blood glucose. Raised TC was defined as TC ≥ 5.0 mmol/L [19].

### Data analysis

Data were entered and coded in accordance with STEPS protocols [19]. Sampling weights were calculated as the inverse probability of selection in the sample, calculated as the product of the probability that each cluster was chosen and the probability that each person from each selected cluster was chosen. Appropriately weighted and stratified estimates of means and proportions, and of regression coefficients, were made using complex survey estimation methods provided by Stata version 14.0 [20].

The associations of various anthropometric measures with continuous outcomes (systolic and diastolic BP, the logarithm of glucose and TC) were estimated by linear regression. To facilitate comparison between estimates, all continuous outcomes and anthropometric indices were transformed to age- and sex-specific z-scores. Poisson regression with robust standard errors [21] was used to estimate prevalence and prevalence ratios of dichotomous outcomes (hypertension, elevated glucose and raised TC).

Logistic regression was used to estimate area under the curve (AUC), which quantifies the capacity of a marker or diagnostic test to discriminate between two groups of subjects [22, 23]. It was used to compare the discriminatory power of BMI and WC and of indices based on them in respect of identifying subjects with hypertension, elevated glucose and raised TC. An AUC of 1.0 indicates perfect positive discrimination, whereas an AUC of 0.5 indicates that the discriminatory power of the predictor is no better than chance alone. All analyses were conducted separately for men and women.

## Results

The characteristics of survey participants are summarised in Table 1. The men on average were heavier and taller than the women, but with similar mean BMI. They also had greater mean WC and hip circumferences, and greater WHR, than the women whose WC relative to height were nevertheless greater than those of the men on average. The men had greater mean AVI than the women, but with similar mean values of BAI, Cindex, and ABSI. The values of BMI and WC were highly correlated for men (r = 0.85) and women (r = 0.77). The men had greater mean systolic and diastolic BP, and the proportion with hypertension was more than 50 percent higher among men than women. The differences in glucose (higher for men on average) and TC (higher from women on average) were not substantial.

**Table 1:** Characteristics of survey participants.

	Men (n = 6 804)	Women (n = 7 902)
	Mean (95%CI)	Mean (95%CI)
Age (years)	40.48 (40.25,40.71)	41.03 (40.80,41.27)
Weight (kg)	56.84 (56.38,57.29)	49.86 (49.56,50.15)
Height (cm)	162.48 (162.25,162.70)	152.41 (152.20,152.63)
BMI(kg/m^2^)*	21.48 (21.35,21.60)	21.49 (21.39,21.60)
WC (cm)	74.78 (74.36,75.20)	71.72 (71.42,72.02)
Hip (cm)	87.83 (87.56,88.10)	86.93 (86.68,87.18)
BAI	-17.58 (-17.58,-17.58)	-17.54 (-17.54,-17.54)
WHR†	0.85 (0.85,0.85)	0.82 (0.82,0.83)
WHtR‡	0.46 (0.46,0.46)	0.47 (0.47,0.47)
AVI	11.52 (11.40,11.64)	10.69 (10.61,10.78)
Cindex	1.16 (1.16,1.17)	1.16 (1.15,1.16)
ABSI	0.08 (0.08,0.08)	0.08 (0.08,0.08)
Systolic blood pressure (mmHg)	124.22 (123.46,124.98)	115.61 (114.97,116.25)
Diastolic blood pressure (mmHg)	75.42 (74.81,76.02)	70.69 (70.21,71.16)
Hypertension (%)	22.15 (20.58,23.73)	14.32 (13.31,15.34)
Glucose (mmol/L)	4.19 (4.13,4.24)	4.04 (4.00,4.09)
Elevated glucose (%)	2.78 (2.05,3.52)	2.62 (2.14,3.19)
Total cholesterol (mmol/L)	4.71 (4.68,4.75)	4.78 (4.75,4.80)
Raised total cholesterol (%)	27.80 (25.88,29.73)	32.27 (30.68,33.86)

* Weight-to-(height)^2^ ratio

^†^ Waist-to-hip ratio

^‡^ Waist-to-height ratio

**Fig 1:**
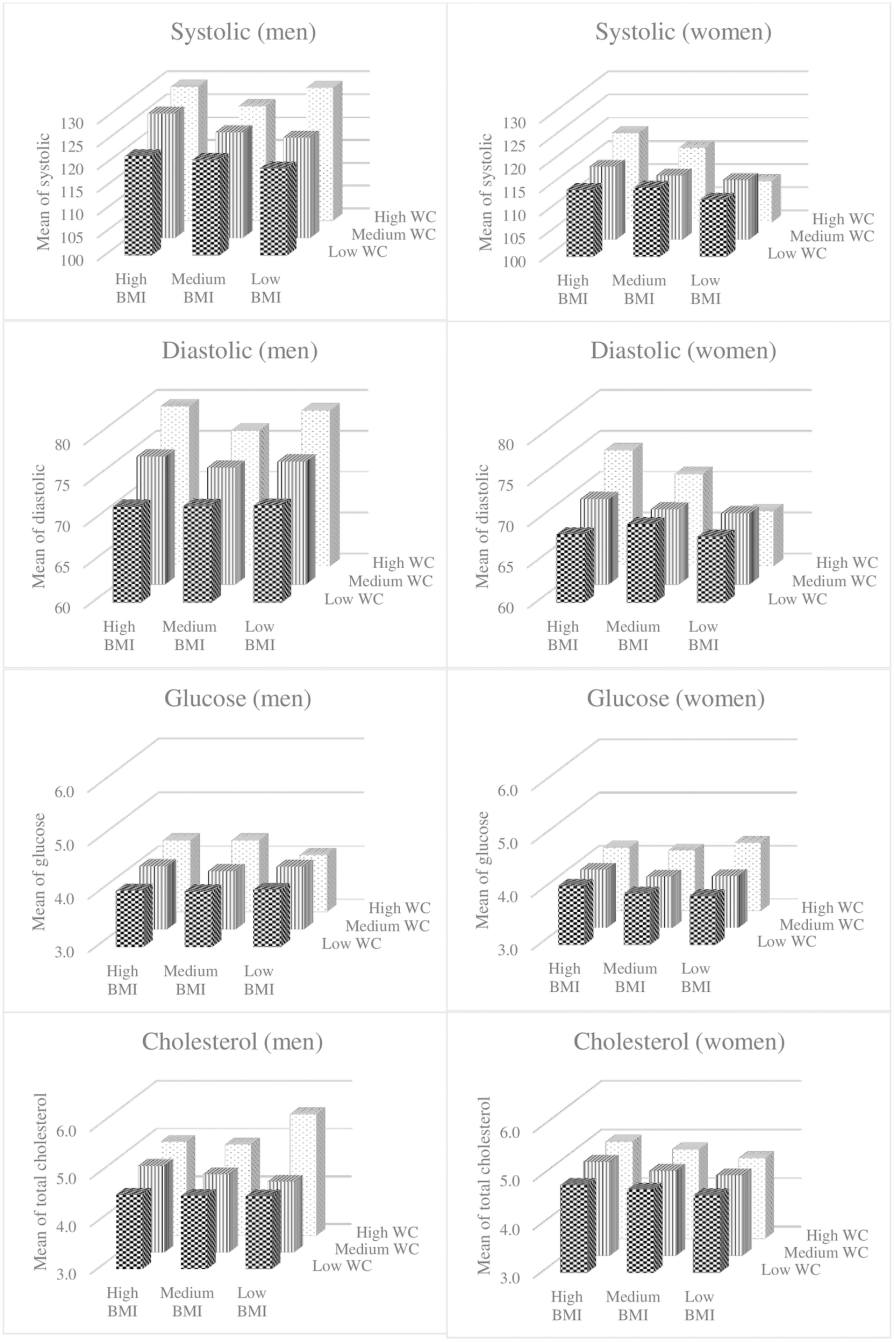
Mean values of systolic BP, diastolic BP, glucose and total cholesterol cross-classified in categories of BMI and WC.

Mean values of systolic BP, diastolic BP, glucose and TC are depicted in Fig 1 for subjects cross-classified by WC and BMI. For this analysis, WC and BMI were each categorised into thirds. For men, the means appear to increase more sharply with WC category than with BMI category. For women, this is true only of glucose concentrations; for BP (systolic and diastolic alike) and for TC, the means appear to increase most sharply with BMI category.

To confirm these observations, and to compare the results for other indices based on weight and girths, Table 2 shows the coefficients from the regression of standardised values of systolic and diastolic BP, the logarithm of glucose concentrations, and of TC concentrations on standardised values of weight, BMI, hip circumference, WC and measures based on these indices. The coefficient of WC or an index comprising WC was the largest in the regressions of each outcome for men, and of log (glucose) for women. For each of the other outcomes for women, the largest regression coefficient was that of either BMI or weight. Because the standardised coefficients are correlation coefficients, it can be inferred that the model R-squared values follow the same pattern: higher for the WC models of men, and higher for the WC model of glucose for women, but otherwise higher for the BMI models for women. Focusing on WC and BMI because these were consistently strong predictors, adjusting one for the other greatly diminished the coefficient of BMI relative to that of WC among men. For women, this was true for glucose but not for systolic BP or TC.

**Table 2:** Coefficients from the regression of standardised values of systolic and diastolic blood pressure, logarithm of glucose, and total cholesterol on standardised values of the anthropometric indices*.

	Systolic BP		Diastolic BP		Glucose		Total cholesterol	
	Men	Women	Men	Women	Men	Women	Men	Women
	β(95%CI)†	β(95%CI)†	β(95%CI)†	β(95%CI)†	β(95%CI)†	β(95%CI)†	β(95%CI)†	β(95%CI)†
Weight	**0.249 (0.19, 0.29)**	**0.189 (0.15, 0.23)**	**0.242 (0.20, 0.28)**	**0.230 (0.19, 0.27)**	**0.114 (0.05, 0.18**	**0.102 (0.06, 0.15**	**0.224 (0.18, 0.26)**	**0.209 (0.17, 0.25)**
BMI	**0.253 (0.20, 0.31)**	**0.198 (0.16, 0.24)**	**0.244 (0.20, 0.29)**	**0.232 (0.19, 0.28)**	**0.121 (0.06, 0.18)**	**0.100 (0.06, 0.14)**	**0.252 (0.21, 0.29)**	**0.236 (0.20, 0.27)**
WC	**0.255 (0.21, 0.31)**	**0.155 (0.12, 0.19)**	**0.284 (0.24, 0.33)**	**0.222 (0.18, 0.26)**	**0.129 (0.07, 0.19)**	**0.104 (0.07, 0.14)**	**0.279 (0.23, 0.33)**	**0.190 (0.15, 0.23)**
Hip	**0.177 (0.13, 0.22)**	**0.105 (0.07, 0.14)**	**0.190 (0.15, 0.24)**	**0.184 (0.14, 0.22)**	**0.095 (0.03, 0.16)**	**0.069 (0.03, 0.11)**	**0.225 (0.18, 0.27)**	**0.167 (0.13, 0.20)**
BAI	**0.144 (0.09, 0.19)**	**0.084 (0.05, 0.12)**	**0.139 (0.09, 0.18)**	**0.143 (0.11, 0.18)**	**0.083 (0.02, 0.15)**	**0.051 (0.02, 0.08)**	**0.211 (0.17, 0.25)**	**0.166 (0.13, 0.20)**
WHR	**0.236 (0.19, 0.28)**	**0.137 (0.10, 0.17)**	**0.269 (0.23, 0.31)**	**0.168 (0.13, 0.20)**	**0.109 (0.06, 0.16)**	**0.096 (0.05, 0.14)**	**0.235 (0.18, 0.29)**	**0.132 (0.09, 0.17)**
WHtR	**0.249 (0.20, 0.30)**	**0.149 (0.11, 0.19)**	**0.270 (0.23, 0.31)**	**0.210 (0.17, 0.25)**	**0.127 (0.06, 0.19)**	**0.099 (0.07, 0.13)**	**0.283 (0.23, 0.33)**	**0.194 (0.15, 0.24)**
AVI	**0.250 (0.19, 0.31)**	**0.152 (0.11, 0.19)**	**0.280 (0.23, 0.33)**	**0.223 (0.18, 0.27)**	**0.134 (0.06, 0.20)**	**0.105 (0.07, 0.14)**	**0.276 (0.23, 0.33)**	**0.176 (0.12, 0.23)**
Cindex	**0.182 (0.14, 0.22)**	**0.053 (0.02, 0.09)**	**0.240 (0.20, 0.28)**	**0.118 (0.08, 0.15)**	**0.099 (0.04, 0.16**	**0.065 (0.02, 0.11)**	**0.238 (0.18, 0.29)**	**0.087 (0.05, 0.13)**
ABSI	**0.099 (0.06, 0.14)**	-0.009 (-0.05, 0.03)	**0.170 (0.13, 0.21)**	**0.048 (0.01, 0.09)**	**0.063 (0.01, 0.11)**	0.035 (-0.01, 0.08)	**0.165 (0.11, 0.22)**	0.019 (-0.02, 0.05)
BMI adjusted WC	**0.128 (0.04,0.21)**	**0.200 (0.14,0.26)**	-0.004 (-0.09,0.08)	**0.167 (0.10,0.23)**	0.037 (-0.04,0.11)	0.052 (-0.03,0.13)	0.041 (-0.04,0.12)	**0.213 (0.16,0.27)**
WC adjusted BMI	**0.144 (0.06,0.23)**	0.005 (-0.06,0.07)	**0.287 (0.21,0.37)**	**0.097 (0.03,0.16)**	**0.098 (0.02,0.18)**	**0.065 (-0.01,0.14)**	**0.244 (0.16,0.33)**	0.031 (-0.02,0.08)

*Data in bold denote statistically significant results.

^†^β(95% CI) = standardised regression coefficient (95% confidence interval)

This effect of mutual adjustment of WC and BMI was even more pronounced when the continuous measures of BP, glucose and TC were replaced by binary measures of high BP (hypertension), elevated glucose and raised TC (see Table 3). For men, the coefficient of WC was not markedly changed on adjustment for BMI but the coefficient of BMI was diminished to near zero. For women, this was true for glucose but, for hypertension and raised TC, the coefficient of BMI was little changed on adjustment for WC whilst that of WC was diminished almost to zero by adjustment for BMI. Not shown in Table 3 is the inconsistent evidence of statistical interaction on the multiplicative scale between BMI and WC, particularly for men. These interactions were negative for hypertension (men p = 0.028) and raised TC (men p = 0.005, women p = 0.028), but positive for elevated glucose (men p = 0.001). The last indicates that the estimated cross-sectional associations of WC and glucose was stronger at higher levels of BMI.

**Table 3:** Coefficients from the regression of hypertension, elevated glucose and raised total cholesterol on standardised values of the anthropometric indices*.

	Hypertension		Elevated glucose		Raised total cholesterol	
	Men	Women	Men	Women	Men	Women
	β(95%CI)†	β(95%CI)†	β(95%CI)†	β(95%CI)†	β(95%CI)†	β(95%CI)†
Weight	**0.262 (0.20, 0.33)**	**0.290 (0.22, 0.36)**	**0.463 (0.28, 0.65)**	**0.251 (0.12, 0.38)**	**0.305 (0.26, 0.35)**	**0.215 (0.17, 0.26)**
BMI	**0.284 (0.21, 0.35)**	**0.309 (0.24, 0.38)**	**0.467 (0.28, 0.65)**	**0.215 (0.09, 0.34)**	**0.329 (0.28, 0.37)**	**0.240 (0.20, 0.28)**
WC	**0.311 (0.24, 0.38)**	**0.246 (0.17, 0.32)**	**0.553 (0.42, 0.69)**	**0.330 (0.23, 0.43)**	**0.350 (0.30, 0.40)**	**0.176 (0.13, 0.22)**
Hip	**0.190 (0.13, 0.25)**	**0.188 (0.12, 0.26)**	**0.306 (0.15, 0.46)**	0.111 (-0.04, 0.26)	**0.265 (0.19, 0.34)**	**0.169 (0.13, 0.21)**
BAI	**0.174 (0.12, 0.23)**	**0.184 (0.12, 0.25)**	**0.260 (0.07, 0.45)**	0.007 (-0.14, 0.15)	**0.245 (0.18, 0.31)**	**0.171 (0.13, 0.21)**
WHR	**0.318 (0.26, 0.38)**	**0.197 (0.13, 0.27)**	**0.638 (0.46, 0.81)**	**0.324 (0.21, 0.44)**	**0.293 (0.23, 0.35)**	**0.116 (0.07, 0.16)**
WHtR	**0.313 (0.25, 0.38)**	**0.246 (0.17, 0.32)**	**0.539 (0.39, 0.69)**	**0.307 (0.21, 0.40)**	**0.352 (0.30, 0.40)**	**0.180 (0.13, 0.23)**
AVI	**0.288 (0.21, 0.36)**	**0.191 (0.12, 0.26)**	**0.523 (0.43, 0.61)**	**0.241 (0.16, 0.32)**	**0.324 (0.27, 0.38)**	**0.142 (0.09, 0.19)**
Cindex	**0.244 (0.19, 0.30)**	**0.123 (0.07, 0.18)**	**0.360 (0.25, 0.47)**	**0.249 (0.16, 0.34)**	**0.257 (0.19, 0.32)**	**0.074 (0.03, 0.11)**
ABSI	**0.171 (0.12, 0.22)**	0.039 (-0.02, 0.10)	**0.296 (0.20, 0.39)**	**0.228 (0.14, 0.32)**	**0.175 (0.12, 0.23)**	0.003 (-0.04, 0.05)
BMI adjusted WC	**0.064 (-0.00,0.13)**	**0.269 (0.19,0.34)**	-0.023 (-0.16,0.12)	-0.038 (-0.14,0.06)	0.107 (0.06,0.16)	**0.235 (0.19,0.28)**
WC adjusted BMI	**0.255 (0.15,0.36)**	0.063 (-0.02,0.14)	**0.569 (0.38,0.76)**	**0.344 (0.21,0.48)**	**0.256 (0.14,0.37)**	0.006 (-0.06,0.07)

*Data in bold denote statistically significant results.

^†^ β(95% CI) = standardised regression coefficient (95% confidence interval)

Finally, the cross-sectional effects of WC or BMI were independent of other cardiometabolic parameters. For example, the regression coefficient of WC in the regression of glucose was reduced by 10.9% (men) or 12.4% (women) by adjustment for systolic BP, and by 10.8% (men) or 10.2% (women) by adjustment for TC.

Table 4 presents AUC in discrimination of hypertension, elevated glucose, and raised TC. Generally, the greatest values of AUC were for WC or an index based on WC, but for raised TC of women, discrimination by BMI was only slightly inferior. The AUC estimates of discrimination were only marginally greater for discrimination by WC and BMI together than for discrimination by WC alone. Other than for raised TC of women, the AUCs for WC within strata of BMI were less attenuated than the AUCs for BMI within strata of WC.

**Table 4:** AUC for discrimination of hypertension, elevated glucose and raised total cholesterol by anthropometric indices.

	Men			Women		
	Hypertension	Elevated glucose	Raised cholesterol	Hypertension	Elevated glucose	Raised cholesterol
Weight	0.598	0.620	0.638	0.622	0.599	0.605
BMI	0.621	0.618	0.657	0.658	0.613	0.635
WC	0.652	0.677	0.669	0.661	0.695	0.628
Hip	0.584	0.589	0.634	0.581	0.536	0.587
BAI	0.591	0.565	0.633	0.616	0.542	0.603
WHR	0.670	0.709	0.645	0.671	0.743	0.618
WHtR	0.660	0.671	0.672	0.675	0.698	0.638
AVI	0.649	0.674	0.669	0.659	0.692	0.627
Cindex	0.653	0.699	0.644	0.625	0.714	0.584
ABSI	0.624	0.685	0.604	0.578	0.686	0.543
BMI + WC	0.652	0.689	0.672	0.668	0.699	0.639
Stratified by WC*						
BMI|Low WC	0.507	0.547	0.564	0.500	0.568	0.575
BMI|Medium WC	0.525	0.573	0.561	0.558	0.519	0.568
BMI|High WC	0.549	0.539	0.546	0.628	0.509	0.579
Stratified by BMI*						
WC|Low BMI	0.615	0.672	0.587	0.556	0.688	0.547
WC|Medium BMI	0.607	0.650	0.599	0.572	0.707	0.559
WC|High BMI	0.602	0.648	0.591	0.600	0.649	0.556

* BMI and WC were divided into thirds

## Discussion

The key finding of this study was that WC or an index based on WC was more strongly associated with BP, glucose and TC for Vietnamese men, and with glucose for Vietnamese women, and provided better discrimination of hypertension, of elevated glucose in particular, and of raised TC for men, than BMI. WC is an indicator of central fat accumulation and the amount of intra-abdominal adipose tissue (IAAT), high levels of which confer an increased risk of cardiometabolic disease [24, 25]. Hence, it might be expected that population data on weight or WC (or an index based on WC such as WHR or WHtR) would be more informative than data on BMI. Whilst BMI is strongly correlated with WC [26, 27], it is a general indicator of excess body weight relative to height, and the correlation of WC with IAAT is greater than that of BMI with IAAT [28]. It is biologically plausible that men have greater central distribution of fat (as indicated by greater WC, WHR, and WHtR) relative to fatmass (as indicated by BMI) than women. In Asian populations as among Caucasians, men are prone to store visceral fat around the abdomen or organs, whereas women typically accumulate fat around the hips, buttocks and thighs [29, 30]. This difference in fat distribution and fat storage can be responsible for different associations of WC and BMI with markers of CVD risk. Our study showed that BMI was a better indicator than WC for predicting BP and TC among women, whereas for men it was the reverse.

WC has been endorsed by several leading national and international organisations as a key indicator of obesity-related health risk [31–33]. This is supported by research findings for both Western [6, 34, 35] and Asian [10, 13, 36] populations. Some previous studies have shown WC to be a stronger predictor and/or better discriminator of CVD risk factors than BMI [37–42]. Others have found that WC performs better for men in United States [34], Japan [43], China [4–46], and Taiwan [47]. WC has been found to be a stronger predictor and/or better discriminator of diabetes for women than BMI [48–50], but BMI performs better for women in Japan [43] and China [44, 45, 51] and Taiwan [47], and for Pima Indians, Native Americans form Arizona [52]. In this nationally-representative sample of Vietnam, WC was more strongly associated with CVD risk for men and of glucose for women, whereas BMI was more strongly associated with BP and total cholesterol among women.

BMI and WC are measured in population studies because they can predict CVD and CVD risk factors. However, each measure has its limitations. For example, WC is not independent of body frame (a tall and athletic person is likely to have a WC that would be considered to be high for a short person). For this reason, some investigators have made use of the WHR and/or WHtR. We investigated four novel indices constructed also using manipulations of general or central obesity measures. These indices were AVI, BAI, Cindex, and ABSI. They allow other dimensions of body size and shape to influence the relationship between BMI or WC and CVD risk factors. Whilst AVI and Cindex provided improved discrimination of elevated glucose relative to BMI, the new indices did not provide stronger prediction or better discrimination than the best-performing traditional indicator.

None of previous hypotheses about the relationships of BMI and WC with cardiometabolic risk is mutually exclusive, and thus their contribution to CVD risk factors is still debated [3]. There is some new understanding provided by this paper in respect of recent debates. Firstly, one issue is whether WC predicts CVD risk as an independent factor. We found that WC independently associated with BP, glucose and total cholesterol for men, and with glucose for women. It provides incremental gains in model fit beyond that provided by BMI. Secondly, another issue is whether the effect of WC on CVD risk is stronger at higher levels of BMI [53]. We found this to be true for elevated glucose in these cross-sectional analyses, but only for men and not for the other outcomes. Thirdly, we found evidence of strong dose–response in cross-sectional associations over most of the entire range of values of WC and BMI, with no evidence of thresholds as some authors have speculated might exist [3, 54–56]. Fourthly, whilst large population-based studies have found strong associations of WC and BMI with cardiometabolic outcomes, the relevant question is whether WC and BMI remains an independent predictor of risk after adjustment for other CVD risk factors. In our study, the estimated cross-sectional effect of WC or BMI was independent of BP and TC in estimation of glucose, of glucose and TC in estimation of BP, and of BP and glucose in estimation of TC. Finally, we found that four recently proposed indicators of risk of CVD and diabetes due to body shape and size (AVI, BAI, Cindex and ABSI) did not improve model fit or subject discrimination relative to the best performing traditional indicator.

The study has several strengths. The data were collected from a nationally-representative survey of the Vietnamese population. The large sample was stratified by sex and rural/urban location, and the availability of data on a range of lifestyle risk factors for non-communicable disease made it possible to take account of confounding factors. To minimise random error and bias, the measurements were made by trained staff in accordance with standardised protocols designed specifically by WHO. We were able to test a wide range of indicators of body size and shape that have emerged from recent research. These included four further indices based on weight, height, hip, and WC, and constructed as ratios of circumference and stature.

However, there are limitations that need to be considered when interpreting the findings of this research. First, while participation was high for a study with overnight fasting and blood sampling, the possibility of non-participation bias cannot be discounted. Second, despite anthropometric and blood pressure measurements with automated equipment in accordance with strict protocols in this survey, measurement errors could have occurred for other reasons (such as faulty recall of medication use in treatment of hypertension or raised blood glucose). Finally, we were restricted to indices based on height and weight and two circumferences, eliminating from consideration those using other circumferences, skinfolds or metabolic parameters.

In conclusion, for these outcomes, WC was more informative than BMI for Vietnamese men, but both WC and BMI were important for Vietnamese women. Both WC and BMI need to be assessed for estimation of CVD risk in Vietnam.
